# Clinical Challenges and Consequences of Measurable Residual Disease in Non-APL Acute Myeloid Leukemia

**DOI:** 10.3390/cancers11111625

**Published:** 2019-10-23

**Authors:** Madlen Jentzsch, Sebastian Schwind, Enrica Bach, Sebastian Stasik, Christian Thiede, Uwe Platzbecker

**Affiliations:** 1 Medical Clinic and Policlinic 1, Hematology and Cellular Therapy, Leipzig University Hospital, 04103 Leipzig, Germany; madlen.jentzsch@medizin.uni-leipzig.de (M.J.); enrica.bach@medizin.uni-leipzig.de (E.B.); 2 Medical Department I, University Hospital and Faculty of Medicine, TU Dresden, 01307 Dresden, Germany; Sebastian.stasik@uniklinikum-dresden.de (S.S.); Christian.thiede@uniklinikum-dresden.de (C.T.)

**Keywords:** AML, measurable residual disease, therapeutic decision-making, risk stratification, pre-emptive therapy

## Abstract

The ability to detect residual levels of leukemic blasts (measurable residual disease, MRD) has already been integrated in the daily routine for treatment of patients with chronic myeloid and acute lymphoblastic leukemia. In acute myeloid leukemia (AML), a variety of mostly retrospective studies have shown that individuals in AML remission who tested positive for MRD at specific time-points or had increasing MRD levels are at significantly higher risk of relapse and death compared to MRD-negative patients. However, these studies differ with respect to the “MRD-target”, time-point of MRD determination, material analyzed, and method applied. How this probably very valuable MRD information in individual patients may be adapted in the daily clinical routine, e.g., to separate patients who need more aggressive therapies from those who may be spared additional—potentially toxic—therapies is still a work-in-progress. With the exception of MRD assessment in acute promyelocytic leukemia (APL), the lack of randomized, prospective trials renders MRD-based decisions and clinical implications in AML a difficult task. As of today, we still do not have proof that early intervention in MRD-positive AML patients would improve outcomes, although this is very likely. In this article, we review the current knowledge on non-APL AML MRD assessment and possible clinical consequences.

## 1. Introduction

Acute myeloid leukemia (AML) is a highly heterogeneous and aggressive neoplasia that is characterized by clonal expansion of myeloid precursors. Over the past few decades, a growing understanding of the molecular landscape and the associated AML biology allowed improved risk stratification at diagnosis. These findings are also reflected in the current World Health Organization (WHO) and European LeukemiaNet (ELN) classifications for AML patients [1,2]. Unfortunately, this progress has not yet led to a substantial improvement of outcome in AML patients. While the majority of AML patients treated with intensive chemotherapy may achieve a morphologic remission, a significant proportion subsequently relapses and dies of their disease [3]. AML relapse is thought to originate from residual cells that survived therapy and that are not detected by conventional morphologic assessments. Novel and more sensitive methods allow for detecting these cell populations, which are referred to as measurable residual disease (MRD) [3]. A perfect MRD test would detect the smallest amount of leukemic blasts that are capable of causing relapse but should be able to discriminate them from healthy (stem) cells or leukemic cells not capable of initiating relapse. A variety of patient or disease related, as well as sampling or test related factors, can lead to reduced sensitivities and specificities and are given in Table 1. Already more than a decade ago, it was demonstrated that the persistence of diagnostic cytogenetic abnormalities in complete remission (CR) associates with adverse outcomes, allowing risk stratification in AML remission [4,5,6]. These days, much effort has been put into the development of new assays, to improve sensitivities and specificities, to optimize standardization between laboratories and to provide applicable techniques for the majority of patients [7,8]. However, the biological heterogeneity, clonal evolution, and the lack of a universal immunophenotype in AML increase the difficulties of establishing standardized MRD assays in AML so far. Recognizing a growing clinical relevance of the MRD status in remission, the ELN defined a “complete remission without minimal residual disease (CR_MRD-_)” as the optimal response category for AML patients [2]. However, today, MRD evaluation in AML is still only routinely used in clinical practice for treatment of acute promyelocytic leukemia (APL) for which standardized guidelines have been published [9,10]. While we still lack data from prospective, randomized clinical trials testing strategies on how to modulate treatment to alter outcomes based on the MRD status in non-APL AML, there is clear evidence that MRD assessment allows outcome prediction [11,12,13,14,15,16]. Subsequently, the most common MRD targets have been increasingly adapted for molecular and flow cytometric MRD assessment in clinical practice. These include the detection of aberrant immunophenotypes by multiparameter flow cytometric (MFC) analyses and the detection of fusion transcripts by quantitative polymerase chain reaction (qRT-PCR) in core-binding factor AML (CBF), as well as *NPM1* mutations in *NPM1*-mutated AML. Here, we review commonly available MRD detection methods in non-APL AML together with their clinical applicability and current practical consequences in daily routines.

## 2. Methods for MRD Detection

After achieving first lasting remissions in AML in the 1960s, for many decades, cytomorphologic assessment with a sensitivity of around 1:20 and high interobserver variability remained the only method to evaluate treatment responses. Later, fluorescence in situ hybridization (FISH) allowed detection of leukemic cells with a sensitivity of up to 1:10^2^ in patients with aberrant cytogenetics. Already this approach allowed risk stratification in remission samples and paved the way for today’s MRD assessments [4,5,6]. Currently, the two most commonly used methods for MRD detection in AML remain MFC and qRT-PCR. Newer technologies represent next generation sequencing (NGS) and digital droplet PCR (ddPCR), which, due to their advantages, will also find broader application in the clinical practice for MRD assessment in the near future. Further comparisons with respect to the commonly available MRD methods and their advantages and disadvantages are given in Table 2.

### 2.1. Multiparameter Flow Cytometry

MFC uses fluorescence labeled antibodies to mark antigens on leukemic (and healthy) cells. It has been integrated in the standard diagnostic work up at AML diagnosis where it allows the differentiation of AML from other diseases, e.g., lymphatic leukemia, but also to define a leukemia-associated immunophenotype (LAIP). This LAIP consists of distinct antigen expression patterns, is unique for the individual AML at diagnosis, and can be tracked as MRD in follow-up samples. A second strategy that is used for MFC-based MRD detection is the “different from normal” (DfN) approach. Here, aberrant immunophenotypic maturation patterns are searched for in AML remission samples. The latter approach does not have an obligatory need for diagnostic material and is also useful to identify new or changed immunophenotypic aberrancies, e.g., due to clonal evolution. Today, the ELN recommends a combination of both approaches; however, as the LAIP also—in most cases—represents a DfN approach, the differences between both methods will most likely diminish over time when larger antigen panels (minimum of eight colors) are used [10]. A clear advantage of MFC-MRD is the applicability in the majority of AML patients (up to 90%). However, MFC-MRD sensitivity is lower than that of most other methods, and it is highly dependent on the degree of difference of the leukemic phenotype to normal antigen expression patterns [17]. Additionally, clonal evolution during the disease course remains a technical challenge as up to 30% of AML patients relapse with an immunophenotype different from the initial clone [18]. Thus, MFC analyses require a high level of experience and the assay quality remains operator-dependent to some degree, hampering inter-laboratory harmonization and standardization. Subsequently, the recent ELN guidelines recommend MFC-MRD analysis to be carried out only in experienced laboratories until techniques have been further standardized [2]. However, the feasibility of MFC-MRD for relapse risk and survival prediction has been extensively published in retrospective analyses for younger and older AML patients. A variety of studies showed an association with higher relapse rates, as well as shorter relapse-free survival and overall survival (OS) for MFC-MRD-positive patients in first morphologic CR after induction chemotherapy [19,20,21,22,23,24,25], after consolidation chemotherapy [20,21,26], after completion of chemotherapy [21], as well as prior to [22,27,28,29,30] or after an allogeneic hematopoietic stem cell transplantation (HSCT) [28].

### 2.2. Quantitative PCR

qRT-PCR represents an established MRD evaluation method that also provides a high sensitivity, relative user-independence, and has—at least to some extent—undergone standardization processes [8,31]. Here, the biggest limitation remains in its restriction to patients harboring specific aberrations, i.e., the core-binding factor (CBF)-AML fusions genes (*CBFB*-*MYH11* in inv(16)(p13q22) or t(16;16)(p13;q22) and *RUNX1*-*RUNX1T1* in t(8;21)(q22;q22)), as well as recurrent mutations, such as *NPM1* mutations. While there is only one known transcript in *RUNX1*-*RUNX1T1,* the three most common breakpoints in *CBFB*-*MYH11* AML [8] and three most common mutations in *NPM1*-mutated AML [32] cover about 90–95% of affected individuals, respectively, and result in assays feasible in clinical practice. Due to this, the ELN recommendations request that patients with one of these alterations should be tested for MRD based on qRT-PCR. However, as the frequency of these aberrations decrease with age, they are applicable in less than 30% of patients over 60 years of age—a population that represents the majority of AML patients [33]. For more infrequent aberrations, patient-specific primer/probe pairs can be designed, but come at a higher cost and lack of data on assay specificity and sensitivity [34]. The sensitivity obtained using qRT-PCR also depends on the target gene and can range from 10^−3^ for *MLLT3*-*KMT2A* [35] up to 10^−6^ for *NPM1* mutated AML [15]. Additionally, inter-individual differences have also been reported [2,33]. For patients lacking leukemia-specific aberrations, the applicability of targeting overexpressed AML-associated genes has been published. Wilm’s tumor gene (*WT1*) is overexpressed in a large proportion of AML patients and *WT1* expression levels have been repeatedly published as potential MRD markers at various time points [36,37,38]. As *WT1* is also expressed in peripheral blood (PB) and bone marrow (BM) of healthy individuals, this physiological background limits the assay’s sensitivity. Thus, the ELN recommends using this marker only in combination or if no other specific marker is available [10]. Expression levels of the leukemia-associated genes brain and acute leukemia, cytoplasmic (*BAALC*) [39,40], and meningioma-1 (*MN1*) [41,42] have also been shown to allow risk stratification in CR, but seem to harbor similar restrictions as *WT1* expression. Another limitation of qRT-PCR remains the need of control-target serial dilutions, which can be overcome by modern methods such as ddPCR assays that also allow a more sensitive MRD detection than qRT-PCR without the need of reference standard urves [40,42,43,44]. As they have also proven robust to variations in PCR efficiency, ddPCR assays represent a promising methodology for MRD monitoring in the future [45].

#### 2.2.1. Core-Binding Factor Leukemia

Due to their excellent response rate to intensive chemotherapy of around 90% and high salvage rate of around 60% in relapsing patients, chemotherapy is usually the chosen consolidation option in CBF-AML. Despite lower assay sensitivity, already 20 years ago, it has been known that assessment of *CBFB-MYH11* and *RUNX1-RUNX1T1* allows risk stratification in CBF-AML patients achieving a CR [46,47]. Since then, a variety of large and/or prospective trials confirmed this knowledge, despite a remaining uncertainty regarding optimal time-points, specimen, and cut-offs that should be used. 

Normalized to 10^5^ *ABL1* copies, after only one cycle of intensive chemotherapy, MRD copy numbers of <100 in BM or <10 in PB for *CBFB-MYH11* AML [16] and <500 in BM or <1000 in PB, as well as a >3 log [16] or >2.5 log [48] BM MRD reduction from diagnosis for *RUNX1-RUNX1T1* AML have been shown to associate with lower relapse risk and longer OS. However, there are also studies not finding a prognostic significance at this early time-point [11,49,50]. After two cycles of chemotherapy, either absolute MRD of <0.1% (normalized to *ABL1*) [50] or a >3 log MRD reduction from diagnosis [48,49,51] associated with a lower relapse risk in both CBF-AML subtypes while MRD-negativity was no prognostic factor in *RUNX1*-*RUNX1T1* AML at this time-point [11]. Normalized to 10^5^ *ABL1* copies, after three cycles of chemotherapy, MRD copy numbers of <10 in PB for *CBFB-MYH11* [16] and <500 in BM, as well as a >4 log BM MRD reduction for *RUNX1-RUNX1T1* AML [16] were prognostic. Finally, in AML with *RUNX1-RUNX1T1,* BM MRD-negativity at the end of treatment was found to associate with OS and disease-free survival (DFS) or relapse probability in some studies [11,48] while another analysis only described a prognostic impact of MRD analyzed in PB [52]. In an analysis by Yin et al. [16], the median time from molecular to morphologic relapse was three months in *CBFB-MYH11* AML and 4.9 and 4.5 months for BM and PB, respectively, in *RUNX1-RUNX1T1* AML [16,52]. The authors suggested that high risk patients with an insufficient decrease of MRD levels might be potential candidates for early allogeneic HSCT in first CR [16,51]. However, there is still no proof from randomized studies that survival can be improved by allogeneic HSCT in these situations [10]. Noteworthy and further complicating reliable recommendations, for both CBF-AML subtypes, some patients with low and stable detection of MRD transcripts had long-term remissions lasting over several years of follow-up—a finding that should be considered when interpreting MRD results in the phase after treatment [16,52,53]. However, MRD monitoring after end of treatment is recommended in CBF-AML patients to detect impeding relapse early [10,16,49,50,51,52,53].

#### 2.2.2. *NPM1* Mutations

*NPM1* mutations are present in approximately 30% of AML patients and are probably among the most evaluated MRD markers in AML, despite a limited comparability due to heterogeneity in the applied assays, tissue used, and time-point of assessment [13,54]. The observation that *NPM1* mutations are present at relapse in nearly all initially *NPM1*-positive patients makes this mutation a reliable MRD marker [13,54]. While *NPM1* MRD analysis in aplasia after induction therapy seems to provide only limited prognostic information [55], for all other time-points, a clinically relevant risk stratification could be shown. After induction and/or consolidation chemotherapy, a <4 log [14] or <3 log [55,56] reduction of *NPM1* transcript levels, as well as still detectable [15] *NPM1* transcripts or levels >0.01% (normalized to *ABL1*) [55] or >0.1 (normalized to *NPM1* wild type copies) [57] were predictive for higher relapse rates, as well as shorter OS and DFS. Additionally, a large analysis by Ivey et al. not only showed PB *NPM1* transcript levels after the second induction therapy to be highly informative for outcome, but also that MRD analysis was superior to diagnostic molecular genetic markers assessed by NGS [13]. Krönke et al. also observed an association between *NPM1* transcript levels and remission duration. Using an arbitrary cut-off of 200 mutated *NPM1*/10^4^ *ABL1*, all patients exceeding this cut relapsed with a median time to relapse of 2.6 months (range 0.4–23.6 months) [15]. As some analyses showed higher values of MRD reduction to baseline and others of absolute thresholds, the optimal cut-off definition remains to be determined but may also be tissue and assay dependent. Analyzing *NPM1* MRD prior to an allogeneic HSCT was also shown to provide prognostic information independently of the used conditioning regimen in a variety of retrospective studies [44,58,59]. Intriguingly, Balsat et al. suggested that *NPM1*^mutated^*FLT3*-ITD^positive^ patients with a >4 log reduction of *NPM1* MRD—but not those with a <4 log reduction—did not benefit from allogeneic HSCT in first CR [14]. Thus, *NPM1* MRD assessment may have the potential to identify patients who benefit from more intensive treatment strategies, which will be further discussed below. Finally, Shayegi et al. observed that, during follow-up, the applied treatment may influence the optimal *NPM1* MRD threshold, as patients whose MRD increased above 1% BM *NPM1*/*ABL1* after chemotherapy, but 10% after allogeneic HSCT had shorter OS and DFS [60].

### 2.3. Next Generation Sequencing

A growing understanding of the genomic landscape in AML led to considerable interest in developing MRD tests using NGS to quantify somatic mutations during disease course. The sensitivity of NGS assays depends on the DNA quality and quantity, as well as the coverage of the sequenced genes. Other limitations arise from clonal heterogeneity with potential outgrowth of small subclones at AML relapse, the intrinsic error rates of NGS platforms, and that detection of mutations is highly dependent on a sufficient depth of sequencing and bioinformative algorithms for mutation calling [61]. Addressing this point, error corrected NGS MRD approaches and exclusion of variants at higher frequencies (e.g., >5%), as they may represent pre-leukemic clones or germline mutations, have been suggested [62]. It remains unclear how to interpret divergent kinetics when assessing numerous mutations together and to discriminate between refractory disease and pre-leukemic clones for some persisting mutations [63]. Additionally, not all mutations in AML associate with equal biologic or clinical consequences if detected in CR. Especially, the clonal hematopoiesis of indetermined potential (CHIP)-associated, so-called DTA mutations (i.e. mutations in the genes *DNMT3A*, *TET2* and *ASXL1*) can reflect pre-leukemic clones rather than residual AML [64,65,66]. Subsequently, the detection of these mutations may provide only limited MRD information, and thus are often excluded from MRD analyses [62,67]. Nevertheless, there is evidence that DTA-mutations might provide MRD information in some situations, e.g., when used in a post-allogeneic-HSCT setting [68]. Despite the still to overcome limitations regarding data sequencing and interpretation, analyzing molecular MRD using NGS already allows identification of patients with a higher relapse risk and shorter OS in the clinical setting of intensive chemotherapy [67,69,70] as well as allogeneic HSCT [62,68,71].

## 3. Practical Challenges

### 3.1. Peripheral Blood or Bone Marrow? 

One major question relates to the optimal specimen for MRD analyses, since the specimen may have great influence on MRD sensitivity irrespective of the applied methodology. In general, 20 mL PB (or more with white blood counts below 1 Gpt/l) or 5–10 mL BM (preferentially the first pull, as PB dilution should be as low as possible) are needed for reliable MRD evaluation [10]. In paired sample analyses, BM repeatedly showed to have higher MRD levels and subsequent higher sensitivity than PB [33]. On the other hand, PB allows a higher convenience for the patient as it spares painful BM aspirations and enables repetitive analyses in shorter intervals which may then provide earlier evidence of MRD recurrence than BM. In general, data on the utility of PB compared to BM remains inconclusive. Numerous studies suggested a somewhat lower MRD burden in PB, but a good correlation between PB and BM MRD, e.g., in MFC [72,73] or qRT-PCR assays for CBF-AML [16,74,75] and *NPM1*-mutated AML [14,15,59] or similar percentages of MRD-positive patients in NGS [61]. This indicates a potential usability of PB in the MRD setting. However, other data showed insufficient correlation disfavoring PB [76], especially in the context of low MRD levels [52]. As a result, especially for MFC-based MRD analyses, PB is not recommended at the moment [10]. An exception remains *WT1* based MRD, where PB seems to be superior to BM analyses due to a higher physiologic *WT1* expression in the latter [31].

### 3.2. Choosing the Optimal Target for MRD Detection

With the introduction of NGS technology, in nearly all individuals, molecular markers may be identified and potentially used for MRD assessment [33,77]. However, the applicability of distinct mutations for MRD analysis is highly variable as some mutations indicate leukemic cells that can initiate relapse while others represent pre-leukemic clones or germline mutations. DTA mutations were shown to be very early events associated with CHIP and can be found in healthy individuals, especially with increasing age (10% of healthy individuals older than 65 years and 18.4% of individuals older than 90 years, but only 1% of those younger than 50 years) [33,78]. In AML patients, these mutations often persist in CR after chemotherapy [13,67,79], and their persistence does not associate with adverse outcomes [67]. In fact, persistence of CHIP-associated mutations might even lead to an enhanced graft-versus-leukemia (GvL) effect and confer a better prognosis in patients undergoing allogeneic HSCT [66,80]. On the other hand, persisting mutations in the genes *NRAS*, *KRAS*, *PTPN11*, and *KIT* associated with worse outcomes, but most likely are later, subclonal events which might lack the high sensitivity wanted for a robust MRD marker [67]. Additionally, germline mutations that confer a higher risk of AML development have been recognized, e.g., in the genes *CEBPA*, *RUNX1* or *GATA2* [81]. As the germline mutational burden will not correlate with the residual leukemic burden, germline origin has to be excluded in cases where these genes are considered for MRD evaluation. The feasibility of MRD targets also depends on the applied therapy, as some MRD analyses are feasible after an allogeneic HSCT. This includes monitoring of donor chimerism in sorted or unsorted PB or BM cells with varying sensitivity [37,82]. Furthermore, monitoring of CHIP-associated mutations e.g., in *DNMT3A*, *TET2*, *ASXL1* [68], or *JAK2* [83] may also provide prognostic information after allogeneic HSCT. The monitoring of the expression levels of AML-associated genes such as *WT1*, *BAALC* or *MN1* may be of help to assess an MRD status in individuals lacking targetable specific aberrations [37,39,40,41,42]. As with MCF-based MRD assessment, expression levels of AML-associated genes are less dependent on certain genetic changes and, thus, have a broader applicability, but are also in general less sensitive. First studies already suggested that combining several MRD targets, e.g., flow cytometry with leukemic stem cell frequency [84] or multiple gene mutations [67] may allow complementary risk stratification. Thus, it is most likely that in the future a combination of MRD targets and assays will be monitored in individual patients, providing a greater certainty to detect residual AML clones capable of initiating relapse.

### 3.3. Timepoint of MRD Evaluation

In general, MRD can be assessed at either “early timepoints“ during therapy cycles to assess kinetics of treatment responses or at “later timepoints“ e.g., after completion of therapy to detect impending morphologic relapse [2]. There are only very limited data on the prognostic significance of MRD assessment at the time-point during aplasia after induction therapy available [85]. For all following time-points during AML therapy—i.e., after induction [19,20,21,22,23,24,25,67,69,70] and consolidation [11,12,20,21,26,52] chemotherapies, prior to [22,27,28,29,30,40,42,45,62,71,86] and after [28,37,68] allogeneic HSCT and during follow up [21]—the predictive value of MRD assessment was extensively published within the last years. However, the optimal timepoints and intervals are yet to be defined and most probably will depend on the underlying AML biology [75]. This is underlined by the fact that heterogeneous kinetics from hematologic to molecular responses, as well as molecular to hematologic relapse have been shown in different AML subtypes [13,15,51,75]. For example, kinetics of *RUNX1-RUNX1T1* and especially *CBFB-MYH1* have been reported to be slower than most other AML subtypes. Here, a molecular relapse can precede hematologic relapse for up to one year [75,87]. In contrast, kinetics for *NPM1* mutated AML seem to be highly variable and dependent on the molecular context, as faster relapses were linked to the presence of a *FLT3*-ITD with a median time to relapse of 3.5 and 6.5 months for *FLT3*-ITD-positive and *FLT3*-ITD-negative patients, respectively [75]. The recently published recommendations from the ELN MRD working party suggest assessing MRD at least at diagnosis, after two cycles of chemotherapy and at the end of treatment in BM and PB, as both specimens have their advantages and disadvantages [10]. Additionally, in patients undergoing allogeneic HSCT, MRD should be evaluated within 28 days prior to the start of the conditioning regimen [10]. During the follow-up phase, MRD should be analyzed every three months in PB and BM for at least two years and according to individual risk thereafter [10].

### 3.4. MRD Thresholds

Similar to the variety of assessable MRD time-points, there is a large heterogeneity regarding the optimal thresholds to define MRD-positive and -negative patients. Different studies made it clear that repetitive detection of high MRD levels, as well as rising MRD levels after molecular remission can reliably predict frank relapse [33,48]. Currently, no MRD test can accurately predict the risk of relapse and defining a threshold for initiation of pre-emptive therapies in MRD-positive individuals remains highly challenging. MRD thresholds probably depend on the applied method and specimen, the analyzed target, and the time-point and context of sampling [88]. Regarding MFC-MRD, most published studies used a cut-off of 0.1% [89]. Consequently, the ELN recommended this cut but also recognized that lower MRD levels still have the potential to cause relapse [10]. For qRT-PCR based MRD analyses, both absolute thresholds (e.g., “negativity”, 0.01% or 0.1%) and log reduction to baseline levels at diagnosis have been identified as clinically relevant. Also for NGS assays, distinct variant allele frequency levels (as negativity, <0.2%, or <2.5%) have been proposed as MRD thresholds [68,69,70,71]. However, in the clinical routine, the sensitivity and threshold applied is most likely of less relevance than the frequency of sampling, the amount, and type of sampled tissue, as well as the clinical interpretation and integration of MRD results into clinical care of patients.

## 4. Clinical Implications of MRD Detection

The repeatedly reported close association between MRD test results and clinical outcome has led to considerable interest in using MRD information for clinical decision-making in everyday therapies. Until today, no randomized studies have been conducted to validate the common suggestion that initiation or intensification of therapies in MRD-positive patients will reduce the risk of hematologic relapse and improve OS in AML patients. The question remains as to whether survival will be truly improved if an individual receives pre-emptive treatment with “only“ a positive MRD test result compared to subjects with frank morphologic relapse [90]. Additionally, the risks of the chosen therapies have to be weighed against the risk of relapse, especially in approaches with higher mortality and morbidity administered to “morphologically cured” AML patients. In AML, the beneficial effect of early treatment intervention has only been convincingly reported for APL patients [9,91,92]. In general, the ELN recommends confirmation of any positive MRD result after 2–4 weeks before judging the relapse probability or starting pre-emptive treatment [10,48,90]. Some prospective non-randomized studies suggested a clinical benefit for MRD-directed therapies in non-APL AML, which are discussed in the following paragraph [14,49,93]. However, as there are no control groups, it remains to be demonstrated that MRD-directed preemptive treatments can actually result in longer survival because also a proportion of patients suffering morphologic relapse can be rescued by chemotherapy and/or allogeneic HSCT [94].

### 4.1. Decision towards Allogeneic Transplant

Allogeneic HSCT is considered to reduce relapse risk at the cost of higher non-relapse mortality and, thus, recommended for patients with higher disease risk in first CR or after relapse. In general, an improvement of DFS of >10% has been adopted to justify the decision towards an allogeneic HSCT [95]. As previously discussed, many studies reported higher relapse incidences and shorter survival for patients being MRD-positive prior to allogeneic HSCT—irrespective of the used MRD marker or intensity of conditioning regimens [22,27,28,29,30,40,42,44,62,71,96]. One study even showed that MFC-MRD-positive patients had outcomes comparable to patients transplanted with active AML [27]. However, despite lacking data from randomized prospective clinical trials, some studies suggested that allogeneic HSCT in MRD-positive patients might improve outcomes. In a cohort of t(8;21) patients, Zhu et al. [49] defined high-risk as failure to achieve a >3 log MRD reduction after the second cycle of consolidation therapy. High-risk patients were recommended to undergo allogeneic HSCT and low-risk patients to undergo consolidation chemotherapy or autologous HSCT. Comparing patients treated according to protocol to patients not treated according to protocol (i.e., low risk patients receiving allogeneic HSCT and high risk patients receiving chemotherapy), this study indicated that allogeneic HSCT significantly reduces relapse rates and improve survival compared to chemotherapy. On the other hand, chemotherapy or autologous HSCT consolidation led to lower relapse rates and longer OS and DFS in low-risk patients. Despite a potential bias introduced through the not-randomized treatment assignment, the study suggested a survival advantage through the risk-adapted treatment approach if comparing the results to data of previous not-risk adapted trials in t(8;21) AML patients [97,98,99,100]. Balsat et al. [14] analyzed mutated *NPM1*-based MRD in PB and defined MRD-negativity as a >4 log reduction. While patients with positive MRD had higher relapse risk and shorter OS, a subanalysis in ELN intermediate risk patients also suggested a favorable impact of allogeneic HSCT on OS in MRD-positive individuals. DFS and OS were similar for MRD-negative patients irrespective of the form of consolidation (allogeneic HSCT or chemotherapy), as well as for MRD-positive patients undergoing allogeneic HSCT. On the other hand, MRD-positive patients consolidated with chemotherapy had significantly shorter DFS and OS [14]. Venditti et al. [101] published a prospective clinical trial where the consolidation therapy (autologous vs. allogeneic HSCT) of younger patients with intermediate genetic risk was dependent on the post-consolidation MRD levels. Despite the non-randomized treatment approach, the authors were able to show that allogeneic HSCT in MRD-positive intermediate risk patients was able to prolong DFS and OS to rates comparable to favorable risk patients. Finally, two other retrospective studies described a favorable impact of allogeneic HSCT compared to chemotherapy alone on relapse rates and OS in patients with positive but not negative MRD in first CR [69,71]. A potential positive impact of allogeneic HSCT in MRD-positive patients is underlined by the observation that GvL effects after HSCT seem to result in similar relative reduction of relapse risk compared to chemotherapy in MRD-positive and MRD-negative patients [102]. The GvL effect might be augmented even further after cord blood compared to BM or PB-derived stem cell transplantation [103,104]. Taken together, these data suggest that, for good- or intermediate-risk AML patients undergoing intensive chemotherapy, MRD evaluation after induction chemotherapy may have the potential to guide decision towards treatment intensification with allogeneic HSCT. Despite the adverse prognostic effect shown for pre-HSCT MRD-positive patients, allogeneic HSCT seems to have the potential to improve outcomes as compared to chemotherapy alone.

### 4.2. Pre-Emptive Treatment of Impeding Relapse

The data on AML patients with molecular relapse or persisting MRD receiving therapeutic intervention are still limited, but experiences from ALL therapy—where MRD triggered treatment with blinatumumab improves outcomes—showed promising results [105]. Also in AML, recent evidence points to an effect of therapeutics, such as azacitidine, to prevent or delay relapse in MRD-positive situations [93]. Within the RELAZA2 trial, AML and high-risk MDS patients in CR prospectively received pre-emptive azacitidine after developing MRD-positive disease defined as either decline of CD34-chimerism <80% or >1% burden of *RUNX1*/*RUNX1T1* or *NPM1* mutation levels. With an acceptable safety profile, azacitidine prevented hematological relapse in 51% of patients. In the remaining patients, relapse could be delayed to a median time of 422 days [93], which was significantly longer than the median 30–120 days reported for historical cohorts [15,75,82]. Additionally, the lower rates of hematologic relapse after allogeneic HSCT compared to relapses after chemotherapy within the study pointed to the immunomodulatory properties of azacitidine [106]. Despite lacking randomized data, this study currently provides the strongest evidence that MRD-guided therapies are able to prevent or substantially delay relapse and—most likely—will prolong survival in MRD-positive AML patients. Within the last years, a lot of new therapeutics targeting AML-associated molecular aberrations such as mutated IDH1 [107], IDH2 [108], FLT3-ITD [109] and/or FLT3-TKD [110], surface antigens [111], or drugs that lead to immunological activation [112] have been evaluated in clinical trials and are currently or will soon be receiving approval. These substances may provide a potential to pre-emptively treat MRD-positive patients at the end of treatment or a possibility to eradicate MRD-positive disease prior to an allogeneic HSCT. Thus, a variety of clinical studies evaluating pre-emptive treatment in patients remaining MRD-positive or suffering molecular relapse are currently enrolling.

## 5. Conclusions

A lot of progress has been made in introducing MRD analyses in the clinical routine, and MRD assessment is already recommended by the ELN for some AML subgroups [10]. Results of MRD testing will probably supplement if not replace the currently used cytomorphology for evaluation of remission after treatment [2] as a variety of studies demonstrated an improved prognostic value of MRD compared to morphological evaluation [19,27]. Regardless of the time-point or MRD target, there is reliable data strengthening the high prognostic significance of MRD assessment. Especially in patients with low or intermediate risk at diagnosis, a repeatedly positive MRD test will probably help to identify patients who benefit from treatment intensification such as an allogeneic HSCT. Additionally, emerging new treatment strategies as targeted therapies may contribute to successful MRD convergence. Despite a growing usage of MRD assessment in the clinical practice, a lot of open questions remain. In the future, protocols should include MRD assessments (including PCR-based and MFC-based approaches) at various meaningful time-points to help with addressing these questions. The most important question to be answered is when and how pre-emptive therapies might improve prognosis in MRD-positive AML patients, which can only be answered by prospective randomized clinical trials. 

## Figures and Tables

**Table 1:** Reasons for inadequate measurable residual disease test results.

Confounding Factors	False Negative Results	False Positive Results
**patient or disease related factors**	- MRD target not consistently present in all AML cells - heterogeneous distribution of leukemic cells in bone marrow - relapse in tissue not assessed, e.g., chloroma or central nervous system	- follow-up time too short or treatment related death - GvL effect after allogeneic HSCT - tested MRD target present on healthy or pre-leukemic cells
**sampling related factors**	- sample size too small - inadequate storing/shipping conditions, too much time from sampling to analysis - PB as used specimen or diluted BM samples	- sample contamination
**assay related factors**	- low assay sensitivity	- external contamination - analysis of dead cells

**Abbreviations:** BM, bone marrow; GvL, graft-versus-leukemia; HSCT, hematopoietic stem cell transplantation; MRD, measurable residual disease; PB, peripheral blood.

**Table 2:** Comparison of different approaches to analyze measurable residual disease (MRD).

Method	Sensitivity	Advantage	Disadvantage
**Conventional Morphology: blast count**	1 in 20 cells	-	-
**FISH: numeric and structural cytogenetic aberrations**	1 in 100–500 cells	- standardized - widely available	- insensitive - limited patients with aberrant karyotype (appr. 50%)
**MFC: LAIP, DfN**	1 in 1000–100,000	- applicable to nearly all AML cases (90%) - can distinguish viable from death cells - short turn around time	- operator-dependent, high experience needed - lower sensitivity and specificity than PCR - difficult to standardize - leukemic phenotype can change over time
**qRT-PCR: molecular aberrations**	1 in 100,000–1,000,000 cells	- high sensitivity - high specificity - existing standardization efforts - operator-independent - short turn around time - widely available	- restricted applicability to patients harboring the specific target (30–50%)
**NGS: molecular aberrations**	1 in 100,000–1,000,000 cells	- high sensitivity - allows to analyze a large number of mutations in a single experiment - easy to perform	- new methodology - currently not widely available - CHIP mutations can be detected in healthy people or may persist in AML remission - limited standardization - intrinsic error rate may limit sensitivity

**Abbreviations:** CHIP, clonal hematopoiesis of indetermined potential; DfN, different from normal; FISH, fluorescence in situ hybridization; LAIP, leukemia-associated immunophenotype; MFC, multicolor flow cytometry; NGS, next generation sequencing; qRT-PCR, quantitative real-time polymerase chain reaction.
